# Association of peripheral and CSF zinc levels with Parkinson's disease: A systematic review and meta-analysis

**DOI:** 10.1016/j.ibneur.2026.02.009

**Published:** 2026-02-12

**Authors:** Rasoul Hossein Zadeh, Reza Hossein Zadeh, Sajjad Hajihosseini, Anahita Rahmati, Yasamin Moeinipour, Omid Salimi, Zahra Rastegar, Ali Molavi, Fateme Sedghi, Nasibeh Zerangian, Mahsa Asadi Anar, Alaleh Alizadeh, Haleh Alizadeh, Niloofar Deravi

**Affiliations:** a Faculty of Medicine, Mashhad University of Medical Sciences, Mashhad, Iran; b Tehran University of Medical Sciences, Tehran, Iran; c School of Medicine, Shahid Beheshti University of Medical Sciences, Tehran, Iran; d Department of Cardiothoracic Surgery, Faculty of Medicine, Mashhad University of Medical Sciences, Mashhad, Iran; e Department of Medicine, Na.C., Islamic Azad University, Najafabad, Iran; f Na.C., Islamic Azad University, Najafabad, Iran; g Department of Psychology, Islamic Azad University, South Tehran Branch, Tehran, Iran; h Ilam University of Medical Science, Ilam, Iran; i Department of Public Health, School of Health, Mashhad University of Medical Sciences, Mashhad, Iran; j Department of Health Education and Health Promotion, School of Health, Mashhad University of Medical Sciences; Student Research Committee, Mashhad University of Medical Sciences, Mashhad, Iran; k Faculty of Medicine, Mashhad Branch, Islamic Azad University, Mashhad, Iran; l School of Pharmacy, Guilan University of Medical Science, Rasht, Iran

**Keywords:** S: zinc, Parkinson's disease, Neurodegeneration, Meta-analysis, Trace element

## Abstract

The possible involvement of numerous chemical elements in the pathogenesis of neurodegenerative diseases has long been studied by researchers, yet no clear consensus regarding the concentration of Zinc (Zn) and the onset of Parkinson's disease (PD) has emerged. The objective of this study was to conduct a robust meta-analysis to clarify the association between Zn levels across different biological matrices and Parkinson’s disease. A comprehensive literature search was conducted across six databases up to April 2024. We included 29 case-control studies reporting Zn concentrations in serum, plasma, and cerebrospinal fluid (CSF) and performed subgroup analyses by biological matrix, continent, and detection method to extend and update previous meta-analyses on this topic. Statistical meta-analysis was performed using STATA v18 software, calculating the weighted mean difference (WMD) and 95 % CIs using a random-effects REML model. Heterogeneity was assessed using the I2 statistic, and publication bias was tested using Begg's and Egger's tests. This meta-analysis pooled data from 29 unique studies. The analysis of Zn in serum (N=22 data points) revealed a statistically significant reduction in PD patients (WMD=−108.23 μg/L, 95% CI: [−205.27, −11.18], p=0.03), with extreme heterogeneity (I²=99.43%). A similar significant deficit was found in plasma (WMD=−258.15 μg/L, 95% CI: [−481.20, −35.11], p=0.02). In contrast, Zn levels in CSF (N=8 studies) showed no statistically significant overall difference (WMD=−15.88 μg/L, 95% CI: [−36.21, 4.46], p=0.13), exhibiting the highest heterogeneity (I2=99.91%). All pooled estimates were characterized by extremely high heterogeneity (I²>99 %), primarily driven by the Asian subgroup and methodological differences, indicating substantial between-study variability and the need for cautious interpretation. Begg’s and Egger’s tests did not suggest substantial publication bias in serum or plasma, but CSF findings should be interpreted with caution. Our research demonstrates a significant association between lower Zn levels in the peripheral circulation (serum and plasma) and susceptibility to PD, suggesting that Zn deficiency may contribute to oxidative stress in PD but without establishing a causal relationship. Our findings reiterate the necessity of large-scale longitudinal cohort studies to validate this association, address the issue of reverse causation, and rigorously evaluate whether correcting Zn deficiency has any therapeutic value in the prevention or progression of PD.

## Highlights

• Systematic review linked zinc levels to Parkinson's disease.
• PD patients show significantly lower blood zinc levels.
• CSF zinc also decreased in Parkinson's patients.
• Geographic variations impact zinc-PD relationship.
• Suggests zinc deficiency as a potential PD risk factor.

## 1 Introduction

Parkinson's disease (PD), originally described by James Parkinson in 1817 and later studied by Jean-Martin Charcot, is a progressively advancing disorder of the central nervous system characterized by motor symptoms such as tremors, rigidity, and bradykinesia (Goetz, 2011, Airavaara et al., 2020). As the disease progresses, patients may also experience difficulty with postural stability. Prevalence of PD has been estimated to be approximately 0.5–1 % in the population aged 65–69 and increased to 1–3 % in the population aged 80 and above (Palakurthi and Burugupally, 2019, Khatib, 2022). Pathologically, PD presents as a decrease in dopaminergic cells that reside in the nigrostriatal tract (Airavaara et al., 2020). However, the neurodegenerative process goes beyond the substantia nigra and inhibits neurons in other neural circuits (Antonina Kouli and Kuan, 2018).

PD has been regarded as having a complex involvement with numerous influences by both genetic and environmental factors despite the elusive identification of its definitive etiology (Abbas et al., 2018, Kalia and Lang, 2015). One such environmental factor suggested to be implicated in PD includes the presence of abnormally elevated concentrations of various heavy metals and metalloids such as zinc (Zn) (Bjorklund et al., 2018, Giacoppo et al., 2014, Vinceti et al., 2018). Some epidemiological studies have revealed a correlation between PD and exposure to heavy metals, which may promote the generation of free radicals through processes such as the Fenton–Haber–Weiss reaction (Ball et al., 2019, Raj et al., 2021, Tchounwou et al., 2012, Vellingiri et al., 2022). Such reactive oxygen species (ROS) would potentially induce oxidative stress and cause mitochondrial dysfunction, DNA breaks, protein misfolding, and eventual neurodegenerative consequences (Vellingiri et al., 2022, Al-Harthi et al., 2022, Lee et al., 2023). On the other hand, various heavy metals such as Zn are crucial trace elements and perform as indispensable cofactors for many enzymes. Alterations in the amounts of heavy metals can negatively impact the nervous system and may play a role in the pathological processes found in PD (Atarod et al., 2022, Fraga, 2005, Tomita and Yoshikawa, 2002, Uversky et al., 2001).

Zn has a critical role in various pathways associated with PD, and beyond its general function as a trace mineral (Chen et al., 2025). As one of the cofactors for Cu/Zn superoxide dismutase, Zn plays a role in protecting against oxidative stress and maintaining mitochondrial function and redox homeostasis within dopaminergic neurons (Palma et al., 2020). Abnormal Zn levels raise α-synuclein aggregation and its interactions with other metal-binding proteins as well as potentially contributing to protein misfolding, oxidative stress, and nerve cell susceptibility in PD (Banik et al., 2025). Both chronic deficiency and excess of Zn can negatively affect the central nervous system; thus, this element has a dual effect on the central nervous system through imbalances (Chen et al., 2025, Nakagawa and Yamada, 2023).

Despite the clear biological importance of Zn status in neurodegeneration, existing observational studies linking Zn concentration to PD have produced inconsistent and conflicting results. This lack of agreement has been emphasized in several previous systematic reviews (Du et al., 2017, Sun et al., 2017, Adani et al., 2020), which reported differing effect sizes, especially across various biological matrices (serum, plasma, CSF) and regions. To address this ongoing uncertainty and provide an updated quantitative summary of this association, we conducted a comprehensive systematic review and meta-analysis. Our study updates earlier reviews by combining a larger amount of recent data and conducting detailed subgroup analyses to examine the impact of biological matrix and geographic origin. In contrast to previous meta-analyses that mainly focused on serum Zn or combined limited matrices, we included serum, plasma and CSF, used weighted mean differences expressed in μg/L, and systematically explored heterogeneity by continent and detection method to extend and refine prior evidence. We aimed to characterize the association between Zn levels in the peripheral circulation and CSF and PD risk, thereby assessing the potential of Zn status as a biomarker and highlighting priorities for future mechanistic and longitudinal studies.

## 2 Methods

### 2.1 Search strategy

Following the Preferred Reporting Items for Systematic Reviews and Meta-Analyses (PRISMA) guidelines (Moher et al., 2009), two independent researchers performed searches through different databases including PubMed, Embase, Cochrane Library, Scopus, Web of Science, and Google Scholar. The articles published up to the 20th of April 2024 were included in the search. A combination of Medical Subject Headings (MeSH) and text terms was employed and listed in Table 1. Moreover, we hand-checked the lists of references in the articles included in our analysis and other relevant reviews and meta-analyses to identify other publications.

**Table 1:** Curated Search strategies for each chosen database and result of the searching procedure.

Data base	Search strategy	results
PubMed	((("Parkinson Disease"[Mesh]) OR ("Parkinson Disease"[Title/Abstract])) OR (parkinson[Title/Abstract])) AND (("Zinc"[Mesh]) OR (zinc[Title/Abstract]))	550
WOS	(TS=("Parkinson Disease")) OR TS= (parkinson) AND TS= (zinc)	663
Scopus	(TITLE-ABS-KEY ( zinc)) AND (TITLE-ABS-KEY (parkinson) OR TITLE-ABS-KEY ("parkinson disease"))	2151

### 2.2 Inclusion/exclusion criteria and study selection

Selection of the articles that were eligible for inclusion relied on a pre-defined set. The inclusion criteria included: being an original research paper for peer review, a human observational study reporting Zn level in serum, plasma or CSF for PD and healthy controls, including English language articles and Parkinson's disease as the outcome. The exclusion criteria included: duplicate publications, studies on animals, and articles lacking original data such as reviews, case reports or abstracts and letters and conference abstracts. We also excluded studies that did not report Zn level for healthy control or whose outcomes were about neuropsychological dysfunction or manganism or parkinsonism or motor dysfunction.

**Fig. 1:**
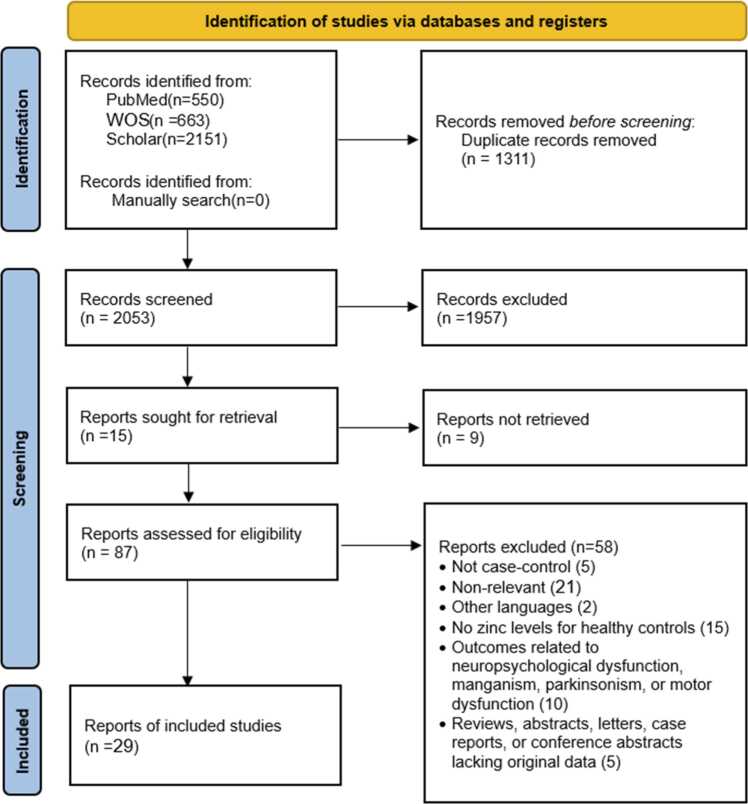
Flow chart of the study selection procedure. Flow diagram illustrating the search strategy and study selection process conducted in accordance with the PRISMA guidelines. The process began with 3364 records and resulted in the final inclusion of 29 unique case-control studies for quantitative synthesis.

Following the elimination of duplicate publications, all articles underwent screening based on their titles and abstracts to filter out irrelevant topics and articles that did not meet the inclusion criteria. Full texts of the remaining articles were obtained and evaluated by one reviewer. Any uncertainties were addressed through discussion with a second reviewer. When multiple publications stemmed from the same study, the most comprehensive and/or latest paper was considered for inclusion. Reanalyses of data from previously published studies that did not provide new insights on the relationship between Zn and Parkinson's disease were excluded. The study selection procedure is illustrated in Fig. 1.

### 2.3 Data extraction

Two authors independently assessed each candidate article and extracted relevant information, including the surname of the first author, publication year, country or region, study design, sample size, age and gender distribution of participants, method of case identification and control selection, and the technique used for Zn level measurement (e.g., AAS, ICP-MS). Additionally, the average Zn levels and their respective standard deviations (SD) were systematically documented, or accurately approximated from median values, ranges, and sample sizes when not explicitly provided.

### 2.4 Risk of bias assessment

Joanna Briggs Institute (JBI) (https://jbi.global/critical-appraisal-tools) critical appraisal checklists were used for evaluating the methodological quality of the articles. Two reviewers independently conducted the quality assessment of all included articles. Any discrepancies were deliberated between the two reviewers, and if a consensus could not be reached, a third reviewer intervened to resolve the disagreement.

### 2.5 Statistical analysis

A meta-analysis was conducted using Zn level data in serum, plasma, or CSF expressed as mean ±SD or standard error of the mean (SEM). The primary outcome was the weighted mean difference (WMD), along with 95 % confidence intervals (CIs). The random-effects model (REML) was utilized to combine the study-specific effect estimates, accounting for the anticipated substantial heterogeneity among studies. Heterogeneity was rigorously assessed using the Chi-square (Q) and I-square (I^2^) tests (Higgins and Thompson, 2002, DerSimonian and Kacker, 2007). A subgroup analysis was performed to investigate the factors contributing to heterogeneity, stratified by continent and detection method. Data points extracted from graphical representations in studies were precisely digitized using WebPlot Digitizer (Automeris LLC, Frisco, Texas) (Rohatgi, 2023). All statistical analyses were performed using STATA statistical software (version 18), with all tests being two-tailed. Statistical significance was predetermined at a p-value < 0.05.

### 2.6 Publication bias assessment

Publication bias was assessed visually using funnel plots and statistically by employing Begg's rank correlation test and Egger's linear regression test (Duval and Tweedie, 2000a, Duval and Tweedie, 2000b, Egger et al., 1997).

### 2.7 Sensitivity analysis

To evaluate the robustness of the overall pooled estimates, a sensitivity analysis was carried out using the one-study-removed method to evaluate the impact of a specific study on the overall estimation of effects (Alexander et al., 2000).

## 3 Results

### 3.1 Study selection and baseline characteristics

Our systematic search process, executed according to the PRISMA guidelines, yielded an initial total of 3364 records across all databases. Following the removal of 1311 duplicate records, the remaining 2053 articles underwent title and abstract screening. This screening step led to the exclusion of 1957 reports. Subsequently, 15 reports were initially sought for retrieval, although this number was later supplemented by hand-checking and other searches, resulting in 87 full-text articles being assessed for eligibility. Of these, 58 reports were excluded due to reasons such as not being a case-control study, lacking Zn levels for healthy controls, being published in other languages, or lacking original data. The detailed exclusion process is depicted in the PRISMA flow diagram (Fig. 1).

The rigorous selection process ultimately identified 29 unique case-control studies that met all predefined inclusion criteria and were included in the quantitative meta-analysis. These studies incorporated data across three biological matrices: serum, plasma, and cerebrospinal fluid (CSF). The baseline characteristics, including country of origin, sample size, age, gender distribution, and Zn detection method for all 29 included studies, are summarized in Table 2. The methodological quality of these studies, assessed using the JBI checklists, ranged from 8 to 10.

**Table 2:** Summarizes the characteristics of the included studies.

**First Author et al. (Year)**	**Specimen Type**	**Continent**	**N-Case**	**Age of Cases (Mean ± SD)**	**Sex in Cases (Female %)**	**Zinc in Cases Mean (SD) (μg/L)**	**N-Control**	**Age of Controls (Mean ± SD)**	**Sex in Controls (Female %)**	**Zink in Controls Mean (SD) (μg/L)**	**Detection Method**	**JBI Score**
Jimenez-Jimenez et al. (1998)	Serum	Spain	37	65.7 ± 8.8	62.16 % (23 F/37 N)	820 (230)	37	62.4 ± 17.8	56.76 % (21 F/37 N)	770(170)	AAS	9/10
Forte et al. (2005)	Serum	Italy	71	65.5 ± 9.4	74.65 % (53 F/71 N)	717.25 (125)	44	51.9 ± 4.0	75.00 % (33 F/44 N)	813(135)	NA	8/10
Qureshi et al. (2006)	Serum	Sweden	17	70 ± 15	58.82 % (10 F/17 N)	910 (70)	21	62 ± 11	61.90 % (13 F/21 N)	890 (20)	AAS	8/10
Gellcin et al. (2008)	Serum	Norway	19	NA	52.63 % (10 F/19 N)	994 (360)	19	NA	51.51 % (51 F/99 N)	1026(323)	ICP-MS	9/10
Nikam et al. (2009)	Serum	India	40	40 −80 (Range)	NA	757.0 (92)	40	NA	NA	985(82.5)	AAS	9/10
Ahmed and Santosh (2010)	Serum	India	45	57.6 ± 9	37.78 % (17 F/45 N)	430 (40)	42	55.6 ± 3.3	45.24 % (19 F/42 N)	590(70)	ICP-MS	
Baillet et al. (2010)	Serum	France	24	60.8 ± 6.5	29.17 % (7 F/24 N)	851.9 (121)	30	60.2 ± 5.1	63.33 % (19 F/30 N)	905(149)	AAS	8/10
Fukushima et al. (2010)	Serum	China	58	63.95 ± 9.40	60.34 % (35 F/58 N)	1150 (470)	81	63.65 ± 9.35	58.02 % (35 F/81 N)	1140(490)	ICP-AES	9/10
Younes-Mhenni et al. (2013)	Serum	Tunisia	48	65.8 ± 10.2	45.83 % (22 F/48 N)	627.6 (170)	36	59.7 ± 12.1	61.11 % (22 F/36 N)	581.9(202.7)	AAS	9/10
Zhao et al. (2013)	Serum	China	238	66.6 ± 11.3	50.84 % (121 F/238 N)	923 (338)	302	65.6 ± 12.2	49.83 % (151 F/302 N)	1293(385)	AAS	9/10
Verma et al. (2016)	Serum	India	225	56.84 ± 8.82	27.11 % (61 F/225 N)	969.12 (301.49)	125	57.24 ± 7.97	28.00 % (35 F/125 N)	1696.67(566.38)	ICP-AES	9/10
Luo et al. (1987)	Serum	China	30	61.5 ± 8.4	0.00 % (0 F/30 N)	812.4(137.3)	30	> 45	0.00 % (0 F/30 N)	909(152.8)	AAS	8/10
Pan (1991)	Serum	China	62	55.4 ± 9.1	37.10 % (23 F/62 N)	673(90.2)	33	52.3 ± 12.8	63.64 % (21 F/33 N)	1269.6(168)	ICP-AES	8/10
Forte et al. (2004)	Serum	Italy	26	64.9 ± 10.8	69.23 % (18 F/26 N)	669(118)	13	63.8 ± 13.7	61.54 % (8 F/13 N)	705(91.1)	ICP-AES	9/10
Hegde et al. (2004)	Serum	India	52	58.1 ± 4.7	46.15 % (24 F/52 N)	457.7(65.4)	25	55.4 ± 6.4	48.00 % (12 F/25 N)	588.4(65.4)	ICP-AES	9/10
Alimonti et al. (2007)	Serum	Italy	71	65.5 ± 9.4	25.35 % (18 F/71 N)	720(78)	124	44.8 ± 12.7	34.68 % (43 F/124 N)	795(88)	ICP-AES	8/10
Brewer et al. (2010)	Serum	US	30	67.4 ± 8.2	40 % (12 F/30 N)	774(94)	29	68.6 ± 6	69 % (20 F/29 N)	827(139)	AAS	9/10
Squitti et al. (2009)	Serum	Italy	28	68.3 ± 11.1	28.57 % (8 F/28 N)	1078(58)	24	66.9 ± 9.5	54.17 % (13 F/24 N)	767(58)	5-Br-PAPS	8/10
Kim et al. (2018)	Serum	Korea	325	65.2 ± 8.7	46.15 % (150 F/325 N)	1438.4(444.6)	304	64.4 ± 9.3	53.62 % (163 F/304 N)	1379.5(438)	ICP-MS	10/10
Barmaki et al. (2021)	Serum	Iran	40	65.70 ± 6.32	32.5 % (13 F/40 N)	725.29(19.23)	40	64.35 ± 3.75	45 % (18 F/40 N)	973.61(15.37)	AAS	9/10
Takahashi et al. (1994)	Serum	Asia	20	61.4 ± 9.3	NR	1817 ± 723	14	60.8 ± 8.9	NR	1540 ± 460	PIXE	9/10
Fang (1994)	Plasma	China	74	56.2 ± 10.8	47.30 % (35 F/74 N)	564(47.47)	66	53.7 ± 14.2	50.00 % (33 F/66 N)	1167.7(48.97)	AAS	9/10
Kocaturk et al. (2000)	Plasma	Turkey	30	64.0 ± 0.0	NA (NA F/30 N)	872.9(130)	24	61.0 ± 0.0	NA (NA F/24 N)	894.6(130)	NA	9/10
Abbott et al. (1992)	Plasma	UK	41	62 ± 9	48.78 % (20 F/41 N)	928.4(94.9)	41	58 ± 9	48.78 % (20 F/41 N)	1222.6(170.8)	NA	9/10
Mcintosh et al. (2012)	Plasma	US	23	70 ± 9	52.17 % (12 F/23 N)	807(56)	24	73 ± 6	58.33 % (14 F/24 N)	805(61)	NA	8/10
Jimenez-Jimenez et al. (1998)	CSF	Spain	37	65.7 ± 8.8	62.16 % (23 F/37 N)	100(60)	37	62.4 ± 17.8	56.76 % (21 F/37 N)	170(140)	AAS	9/10
Qureshi et al. (2006)	CSF	Sweden	17	70 ± 15	58.82 % (10 F/17 N)	105.92 (19)	21	62 ± 11	61.90 % (13 F/21 N)	161 (31)	ICP-AES	9/10
Alimonti (2007)	CSF	Italy	42	64.5 ± 10.7	52.38 % (22 F/42 N)	2.77(0.9)	20	66.2 ± 14.7	70.00 % (14 F/20 N)	2.23(1.14)	ICP-MS	9/10
Hozumi et al. (2011)	CSF	Japan	20	68.7 ± 5.8	55.00 % (11 F/20 N)	14.5(7.6)	15	48.4 ± 22.2	60.00 % (9 F/15 N)	5.3(3.3)	ICP-MS	10/10
Takahashi et al. (1994)	CSF	Japan	19	61.4 ± 9.3	NA	258 (182)	14	60.8 ± 8.9	NA	420 (350)	PIXE	9/10
Sanyal et al. (2016)	CSF	India	50	58.7 ± 12.4	32.00 % (16 F/50 N)	687.4(81.1)	60	60.1 ± 10.4	30.00 % (18 F/60 N)	700.2(86.7)	ICP-MS	9/10
Maass et al. (2018)	CSF	Germany	36	65.5 ± 13.1	33.33 % (12 F/36 N)	9.48(5.9)	42	67 ± 11	42.86 % (18 F/42 N)	14.22(25.72)	NA	9/10
Willkommen et al. (2018)	CSF	Germany	33	65.1 ± 12.9	30.30 % (10 F/33 N)	18.98(3.19)	101	44.8 ± 17.3	62.38 % (63 F/101 N)	15.8(0.05)	NA	10/10

### 3.2 Zinc concentration meta-analysis

The meta-analysis was performed using the WMD (μg/L) for Zn levels across three matrices to quantify the association between Zn status and Parkinson’s disease (PD) susceptibility.

#### 3.2.1 Zinc in serum

**Fig. 2:**
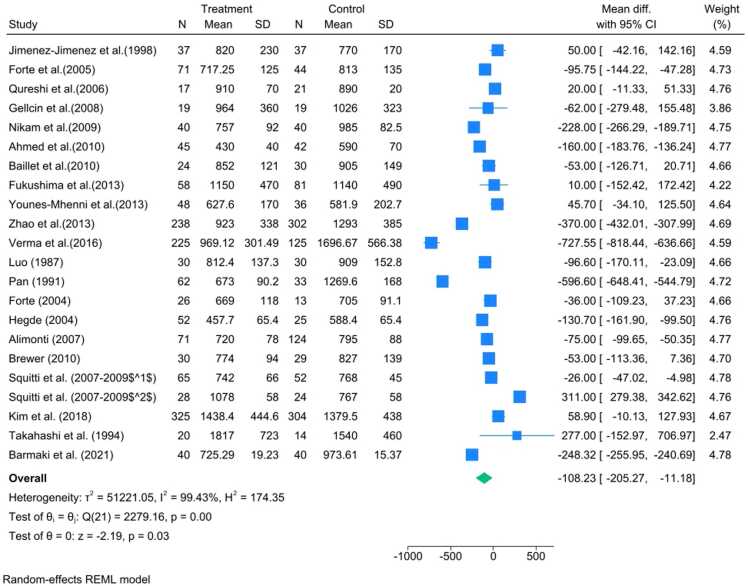
Forest plot of Zinc levels in serum for Parkinson's disease (PD) patients versus controls. Forest plot displaying the Weighted Mean Difference (WMD) in serum Zinc concentration (μg/L) for individual studies and the pooled estimate. The overall analysis showed a significant reduction in PD patients (WMD=−108.23 μg/L, 95% CI: [−205.27,- 11.18], p=0.03). The analysis was characterized by extreme heterogeneity (I2=99.43%). The diamond represents the pooled effect size under the random-effects REML model.

The pooled results for serum Zinc (N=22 data points) revealed a statistically significant reduction in PD patients when contrasted with healthy controls. The overall effect demonstrated a WMD of −108.23 μg/L (95% CI:[−205.27,−11.18]), with a high level of significance (p=0.03). This finding was accompanied by extreme heterogeneity (I2=99.43%), indicating considerable variance across the studies included (Fig. 2).

**Fig. 3:**
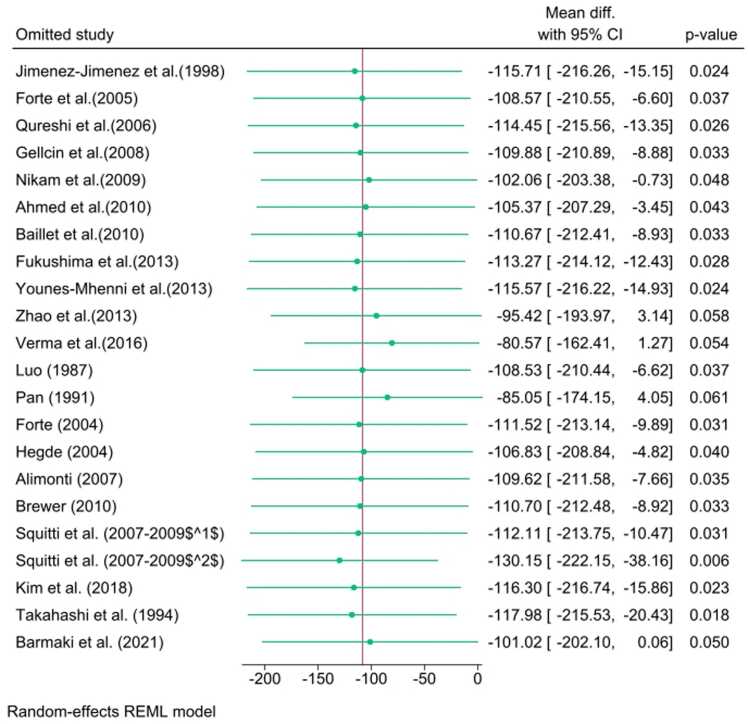
Sensitivity analysis of serum Zinc levels using the one-study-removed method. Sensitivity plot showing the recalculated pooled WMD in serum Zn concentration after iteratively excluding each study. The plot demonstrates the robustness of the overall finding, as the pooled effect remained statistically significant and consistently negative regardless of which single study was omitted.

Subgroup analysis was performed to investigate potential sources of this high heterogeneity. Stratification by continent indicated that the significant Zinc deficit was primarily driven by the Asian subgroup (WMD=−220.69 μg/L, p=0.006). Furthermore, stratification by detection method confirmed a significant reduction in studies utilizing AAS, NA and ICP-AES (Fig. 3).

**Fig. 4:**
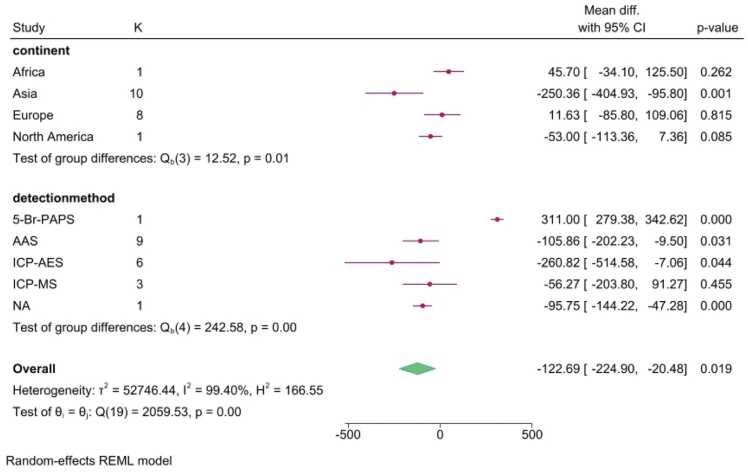
Subgroup plot illustrating the effect of different covariates on the WMD in serum Zinc levels. The analysis confirmed that geographical location was a significant source of heterogeneity, with the Asian subgroup showing the most significant deficit (WMD=−220.69 μg/L, p=0.006). Significant differences were also noted across analytical detection methods (AAS, ICP-AES, and NA

Sensitivity analysis, conducted using the one-study-removed method, confirmed the robustness and stability of the overall pooled estimates across all three matrices. The sequential omission of any single study did not significantly alter the primary conclusion of reduced Zn levels in the serum (Fig. 4).

#### 3.2.2 Zinc in plasma

**Fig. 5:**
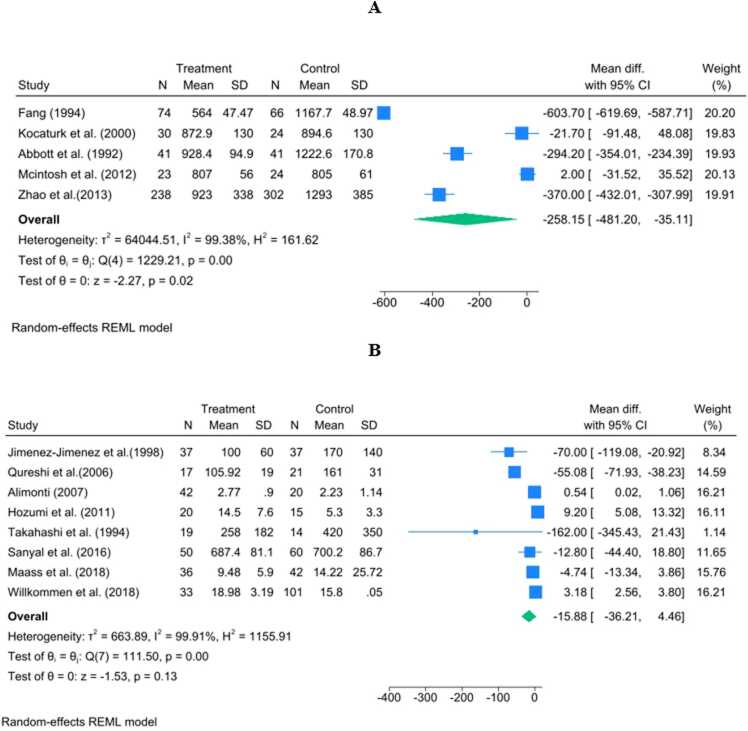
Forest plots of Zn levels in the plasma and CSF matrices. Forest plots summarizing the WMD in Zn concentration (μg/L) for the plasma and CSF matrices in PD patients versus controls. Panel A shows plasma Zn, with a significantly lower level in PD patients (WMD = −258.15 μg/L, 95 % CI: [−481.20, −35.11], p = 0.02) across four studies. Panel B shows CSF Zn, where no statistically significant overall difference was observed between PD patients and controls (WMD=−15.88 μg/L, 95% CI:[−36.21,4.46], p=0.13) across eight studies.

The analysis of the plasma matrix (N = 4 studies) corroborated the serum findings, showing a deeper and statistically significant reduction in Zn levels among PD patients. The pooled WMD was −258.15 μg/L (95 % CI:[−481.20,−35.11]), with a significant overall effect (p = 0.02). This analysis was also characterized by extremely high heterogeneity (I2 =99.38 %), primarily reflecting the substantial discrepancies in effect size among the limited number of contributing studies (Fig. 5A).

#### 3.2.3 Zinc in cerebrospinal fluid (CSF)

The combined findings for Zinc levels in CSF (N=8 studies) revealed no statistically significant overall difference between PD patients and healthy controls. The pooled WMD was −15.88 μg/L (95% CI:[−36.21,4.46]), with a non-significant p−value of 0.13. This analysis exhibited the highest level of heterogeneity (I2=99.91%), resulting from the severe directional conflicts reported by individual studies (Fig. 5B).

### 3.3 Publication bias and sensitivity

Sensitivity analysis, carried out using the one-study-removed method, showed that excluding any single study did not significantly alter the pooled analysis, confirming the robustness of the overall findings across all three matrices.

**Fig. 6:**
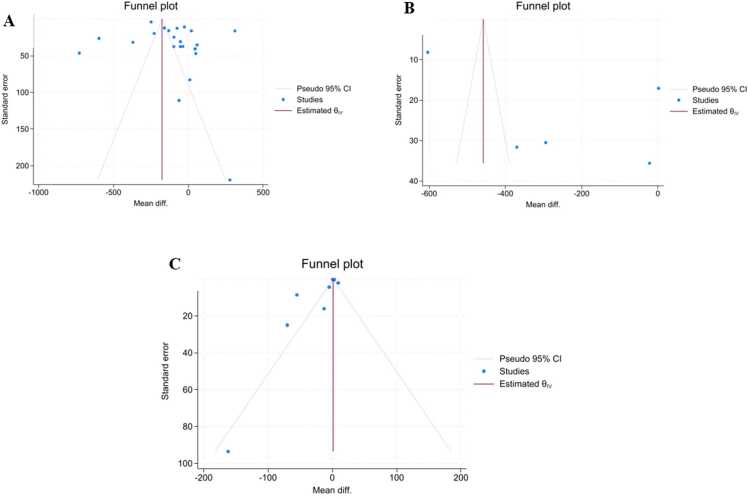
Funnel plots illustrating publication bias in the meta-analysis of Zinc levels. Funnel plots visually assessing publication bias for the three biological matrices in the meta-analysis of Zn levels. Panel A shows serum, panel B plasma and panel C CSF. The relative symmetry observed in the funnel plots, together with the formal tests, did not indicate substantial publication bias overall, although the CSF findings should be interpreted cautiously.

Publication bias was evaluated using Begg's test and Egger's test, and visually inspected via funnel plots (Fig. 6). Overall, the funnel plots and formal tests did not indicate substantial publication bias, although the Egger’s test result for CSF (p=0.0029) should be interpreted cautiously in light of the limited number of studies and extreme heterogeneity:

• Serum: Begg's p=0.4298; Egger's p=0.2951.
• **Plasma:** Begg's p = 0.8065; Egger's p = 0.3751.
• **CSF:** Begg's p=0.5362; Egger's p=0.0029.

## 4 Discussion

The principal finding of this comprehensive meta-analysis is the consistent and statistically significant reduction in Zn concentration in the peripheral circulation (serum and plasma) of Parkinson's disease (PD) patients compared to healthy controls. Specifically, the pooled WMD of −122.69 μg/L in serum and −258.15 μg/L in plasma demonstrates a substantial systemic deficit, supporting the hypothesis that lower Zn levels are associated with PD and may contribute to oxidative stress, while not establishing a causal role in the disease’s pathogenesis. The strength of this association is corroborated by the absence of significant publication bias in all three matrices (serum: Begg's p = 0.6732, Egger's p = 0.8329).

However, the interpretation of the overall effect is significantly complicated by the extreme heterogeneity (I2 >99 %) observed across all three matrices. Our analysis indicated that only geographical location had a significant impact on this high heterogeneity. Subgroup analysis revealed that the statistically significant Zn deficit was overwhelmingly concentrated in Asian nations (WMD=−250.36 μg/L, p = 0.001), while Western countries did not demonstrate any significant overall relationships. This geographical variation aligns with the findings of other independent meta-analyses, suggesting that regional differences in dietary Zn intake, environmental exposures, or genetic background are pivotal sources of the observed discrepancies. Furthermore, the high heterogeneity was amplified by methodological variances; the significant WMD reductions from AAS and ICP-AES were countered by an opposing, significant increase reported by the single outlier study using the 5-Br-PAPS method (WMD=311.00 μg/L).

In stark contrast to the clear peripheral deficit, the meta-analysis of CSF Zn levels found no statistically significant overall difference (WMD=−11.96 μg/L, p = 0.23). This non-significant finding, coupled with the highest heterogeneity (I2 =99.91 %), is particularly challenging. The profound directional conflict among individual CSF studies—some reporting significant decreases (Qureshi et al., 2006, Jimenez-Jimenez et al., 1998), others reporting significant increases (Alimonti et al., 2007, Hozumi et al., 2011) —suggests a complex Zn compartmentalization issue in the CNS rather than a simple global deficit. This indicates that the peripheral status may not accurately mirror the micro-environmental balance of Zn across the Blood-Brain Barrier (Qi and Liu, 2019).

The observed peripheral Zn deficiency is mechanistically supported by Zn's role as a potent anti-oxidant (Kiouri et al., 2023). Zn is a primary cofactor for antioxidant enzymes such as Superoxide Dismutase (SOD), catalase, and peroxidase. Decreased Zn levels compromise the activity of key antioxidant enzymes like Cu/Zn SOD and reduce Zn metallothionein activity, thereby inducing oxidative stress and elevating free radicals, which leads to cellular deterioration and necrosis in dopaminergic neurons (Sofo et al., 2018, Mariani et al., 2008, Kavian et al., 2022). Additionally, Zn inhibits the production of ROS by competing with redox-active metal ions like Copper (Cu) and Iron (Fe) for binding sites on cellular macromolecules (Qureshi et al., 2006). The age of the PD patient is also a primary complicating factor; our meta-analysis revealed that all included studies, with patient mean ages ranging from 59.3 to 71.66 years, were significantly impacted by age, suggesting a potential role for age in the outcome of heterogeneity (McMillan et al., 2021). Beyond these antioxidant effects, dysregulated Zn homeostasis can modulate α-synuclein aggregation and its interaction with metallothioneins and other metal-binding proteins, potentially influencing mitochondrial dysfunction and neuronal vulnerability in PD (Chen et al., 2025, Nakagawa and Yamada, 2023).

This comprehensive meta-analysis significantly strengthens the evidence for a systemic Zn deficit in PD patients, largely mirroring and extending the findings of previous major meta-analyses. For instance, Du et al (Du et al., 2017). reported a significant reduction in serum Zn (SMD=−0.59) and a reduced Zn level when combining serum and plasma data (SMD=−0.60), results that align closely with our own observation of a statistically significant WMD in both serum and plasma. Similarly, Sun et al (Sun et al., 2017)., focusing solely on serum, found a significant SMD of −0.779 and also highlighted the strong effect observed in Asian studies, which we meticulously confirmed and quantified through our detailed subgroup analysis (WMD=−250.36 μg/L). Our study contributes to this body of work by providing results expressed in μg/L (WMD) from a larger number of studies, offering a more clinically interpretable metric than the SMD used in many earlier reports.

In addition to the corroboration of earlier meta-analyses, there are a number of new contributions made by our work. First, we included a greater number of case-control studies and provided evaluations as of April 2024. Secondly, all studies evaluated Zn in the form of plasma, serum and CSF together to facilitate more clinically relevant pooled effect estimates (WMD expressed as μg/L). Lastly, we were able to show that deficits in peripheral Zn levels are primarily attributed to Asian populations and to certain types of analytical methods through pre-specified subgroup analyses of cases and controls conducted by continent and Zn detection method, and thus we refined previous estimates and produced new hypotheses through the introduction of an increased number of case-control studies and the inclusion into the dataset of other variables associated with peripheral Zn levels.

A crucial point of comparison and innovation in our study lies in the detailed analysis of the CSF compartment, a feature also explored by Adani et al (Adani et al., 2020). While Du et al (Du et al., 2017) observed a non-significant trend toward reduced CSF Zn levels, the contradictory findings and the high I2 (99.91 %) in our analysis demand a more cautious interpretation: the core issue is likely related to dysregulated transport of Zn across the Blood-Brain Barrier (BBB), where Zn transporters ZnTs and ZIPs may be impaired, rather than a simple CNS deficiency. This finding is consistent with the general conclusion of Adani et al. (2020) that methodological factors, including the type of biological sample and detection technique, strongly influence the heterogeneity and final pooled estimates. While peripheral Zn levels seem to be a consistent risk indicator at the systemic level, how CNS Zn is affected remains highly debated and may depend on regional compartmentalization and disease stage. Future studies using advanced imaging or post-mortem analysis are necessary to accurately map Zn distribution and understand the role of Zn-regulating proteins in Parkinson's disease brains. Although our results indicate a systemic Zn deficit in peripheral circulation, maintaining overall Zn balance is crucial, as both deficiency and excess can be detrimental to neuronal integrity. It's equally important to investigate the impacts of Zn overload, which can lead to neurotoxicity and the production of reactive oxygen species. The diverse and often conflicting data in CSF may partly stem from local regulatory issues caused by Zn deficiency or excess within the central nervous system environment.

The results of our meta-analysis are robust at the statistical level, with the sensitivity analysis showing that excluding any single study did not materially change the overall findings. However, we must acknowledge that all studies were case-control investigations (with only one being prospective), which are inherently susceptible to reverse causation. The observed Zn deficit may partly reflect consequences of PD itself like impaired nutritional status or disease-related metabolic changes),rather than a primary etiological risk factor. This potential for bias, along with the lack of detailed data on key confounding factors like disease stage, duration, drug use, and exact gender distribution in the existing literature, necessitates caution in interpreting causation. The limitation of only including English-language studies also introduced selection bias. Further study using robust longitudinal design methodologies will be required to confirm our current results definitively.

## 5 Conclusion

In conclusion, our evidence demonstrates a consistent association between decreased Zn levels in the peripheral circulation (serum and plasma) and Parkinson’s disease susceptibility, although substantial between-study heterogeneity warrants cautious interpretation. These findings are compatible with a potential contribution of Zn deficiency to oxidative stress in PD, but they do not establish a causal role for Zn imbalance in PD pathogenesis. Our findings reiterate the necessity of large-scale longitudinal cohort studies to validate this association, clarify the direction of causality, and rigorously evaluate whether correcting Zn deficiency has any therapeutic value in the prevention or progression of PD.

## List of abbreviations

**AAS**: Atomic Absorption Spectrometry
**BBB**: Blood-Brain Barrier
**CI**: Confidence Interval
**CNS**: Central Nervous System
**CSF**: Cerebrospinal Fluid
**Cu**: Copper
**Fe**: Iron
**I**^**2**^: I-square test (Heterogeneity statistic)
**ICP-AES**: Inductively Coupled Plasma-Atomic Emission Spectrometry
**ICP-MS**: Inductively Coupled Plasma-Mass Spectrometry
**MeSH**: Medical Subject Headings
**NOS**: Newcastle-Ottawa Scale
**PD**: Parkinson's Disease
**PRISMA**: Preferred Reporting Items for Systematic Reviews and Meta-Analyses
**Q**: Chi-square test (Heterogeneity statistic)
**REML**: Restricted Maximum Likelihood
**ROS**: Reactive Oxygen Species
**SD**: Standard Deviation
**SEM**: Standard Error of the Mean
**SMD**: Standardized Mean Difference
**SOD**: Superoxide Dismutase
**WMD**: Weighted Mean Difference
**Zn**: Zinc

## Ethical approval

This systematic review and meta-analysis of previously published studies did not involve human participants or animal subjects. Therefore, an ethical review was not required.

## Compliance with ethical standards statement

This systematic review and meta-analysis involved no direct human or animal experimentation, and all data was extracted from previously published studies. Therefore, ethical approval and informed consent were obtained by the original study authors, as per their respective institutional guidelines.

## Sources of support

This research did not receive any specific grant from funding agencies in the public, commercial, or not-for-profit sectors.

## CRediT authorship contribution statement

**Ali Molavi:** Writing – original draft. **Omid Salimi:** Writing – original draft. **Zahra Rastegar:** Writing – original draft. **Mahsa Asadi Anar:** Writing – original draft. **Alaleh Alizadeh:** Writing – review & editing, Supervision, Project administration, Conceptualization. **Rasoul Hossein Zadeh:** Writing – original draft. **Fateme Sedghi:** Writing – original draft. **Nasibeh Zerangian:** Writing – original draft. **Anahita Rahmati:** Writing – original draft. **Yasamin Moeinipour:** Writing – original draft. **Haleh Alizadeh:** Writing – review & editing. **Reza Hossein Zadeh:** Writing – original draft. **Niloofar Deravi:** Writing – review & editing. **Sajjad Hajihosseini:** Writing – original draft.

## Declaration of Generative AI and AI-assisted technologies in the writing process

AI was used to paraphrase the text.

## Declaration of Competing Interest

The authors declare that they have no known competing financial interests or personal relationships that could have appeared to influence the work reported in this paper.

## Data Availability

The authors confirm that the data supporting the findings of this study are available within the article and its supplementary materials.
